# cAMP Is a Promising Regulatory Molecule for Plant Adaptation to Heat Stress

**DOI:** 10.3390/life12060885

**Published:** 2022-06-14

**Authors:** Shuang Liang, Jinfeng Sun, Yanmin Luo, Shanshan Lv, Jiajia Chen, Yanpei Liu, Xiuli Hu

**Affiliations:** State Key Laboratory of Wheat & Maize Crop Science, College of Life Science, Henan Agricultural University, Zhengzhou 450002, China; 15210659252@163.com (S.L.); 17703811466@163.com (J.S.); l13346631399@163.com (Y.L.); henaulvss@163.com (S.L.); 13592663987@163.com (J.C.)

**Keywords:** cAMP, heat stress, heat response, plants, ABA, Ca^2+^

## Abstract

With gradual warming or increased frequency and magnitude of high temperature, heat stress adversely affects plant growth and eventually reduces plant productivity and quality. Plants have evolved complex mechanisms to sense and respond to heat stress which are crucial to avoiding cell damage and maintaining cellular homeostasis. Recently, 33″,55″-cyclic adenosine monophosphate (cAMP) has been proved to be an important signaling molecule participating in plant adaptation to heat stress by affecting multi-level regulatory networks. Significant progress has been made on many fronts of cAMP research, particularly in understanding the downstream signaling events that culminate in the activation of stress-responsive genes, mRNA translation initiation, vesicle trafficking, the ubiquitin-proteasome system, autophagy, HSPs-assisted protein processing, and cellular ion homeostasis to prevent heat-related damage and to preserve cellular and metabolic functions. In this present review, we summarize recent works on the genetic and molecular mechanisms of cAMP in plant response to heat stress which could be useful in finding thermotolerant key genes to develop heat stress-resistant varieties and that have the potential for utilizing cAMP as a chemical regulator to improve plant thermotolerance. New directions for future studies on cAMP are discussed.

## 1. Introduction

cAMP is the first discovered second messenger. Adenylyl cyclases (ACs) are responsible for catalyzing ATP to generate cAMP, which is further degraded by phosphodiesterases (PDEs). cAMP has long been proved to play vital roles in a wide variety of physiologic responses in animals, algae, fungi and bacteria [1,2].

All known eukaryotic ACs belong to the ubiquitous Class III. In mammalian cells, all AC isoenzymes (AC1–10) belong to AC Class III according to sequence homology in the catalytic domain, but can be separated into two distinct types: nine transmembrane enzymes (tmAC; AC1–9) and one soluble AC (sAC; AC10) [1,2,3]. tmACs are mainly regulated by heterotrimeric G-proteins, which are key signalling switches of the G-protein coupled receptor pathways, while sAC is directly activated by Ca^2+^ and bicarbonate (HCO_3_^−^), and acts as a sensor for ATP, Ca^2+^, and CO_2_/HCO_3_^−^/pH at various intracellular locations [4,5]. Additionally, at least four types of cAMP effectors have been found in mammalian cells: protein kinase A (PKA), cyclic nucleotide gated ion channels (CNGs), exchange proteins activated by cAMP (EPACs) which is also known as the cAMP-regulated guanine nucleotide exchange factor (cAMP-GEFs), and PDEs. Notably, in the cAMP-PKA signaling pathway, cAMP activates PKA which then phosphorylates many kinases such as GSK3, Raf and FAK [1,2,3,4,5]. An aberrant cAMP-PKA signaling pathway often leads to various types of human diseases. In the EPACs-mediated signaling pathway, the significance of each EPAC in different cell systems is extraordinary. The study of EPACs has substantially expanded the diversity and adaptive nature of cAMP signaling relating to numerous pathophysiological and physiological responses [1]. The above-mentioned perspectives have been well-reviewed.

By contrast, comprehensive knowledge of cAMP signal transduction in higher plants has yet to be fully clarified. Over the last twenty years, several studies about the biological functions of ACs and their catalytic product cAMP have been reported in plants. Particularly in plant biology research, recent advances supported by biochemical, genetic and omics studies have proven that cAMP, as a polyhedral molecule, is critically involved in the signaling pathways of both plant development and response to environmental stress [6,7,8,9,10].

Since the publication of the first identification of the *AC* gene in plants [11], much effort has been put into demonstrating new *ACs* in plants during the past 21 years because of its crucial functions in other organisms [1,2]. To our knowledge, eleven *AC* genes in plants have been reported: two *ACs* in *Zea mays* i.e., *ZmPSiP* [11] and our newly identified *ZmRPP13-LK3* [9]; six *ACs* in *Arabidopsis* i.e., *AtPPR-AC* [12], *AtLRRAC1* [13], *AtKUP5* [14], *AtKUP7* [15], *AtClAP* [16] and *crypto-AC* [17]; an *NbAC* in *Nicotiana benthamiana* [18]; an *HpAC1* in the *Hippeastrum × hybridum* [19]; and an *MpCAPE* in the liverwort *Marchantia polymorpha* [20]. However, the knowledge about these *ACs* and their catalytic product cAMP remains poorly understood.

At present, ACs and their catalytic product cAMP, which functions as the regulatory components of many plant responses, including plant growth, development, and response to stress, have been discussed in various reviews under normal or stress conditions [6]. Nevertheless, the role of cAMP in adaptation to heat stress has not been extensively summarized. This review gives an overview of the existing information about the upstream and downstream effectors of ACs-cAMP signaling cascades in plants; furthermore, the potential strategies to target the ACs-cAMP pathway for plant adaptation to heat stress are discussed, facilitating the establishment of a new model of ACs-cAMP regulation in plants.

## 2. Diversity Features of the Catalytic Center Motifs in the Identified Plant ACs

In plants, eleven *AC* genes have been identified, including *ZmPSiP* [11], *ZmRPP13-LK3* [9], *AtPPR-AC* [12], *AtLRRAC1* [13], *AtKUP5* [14], *AtKUP7* [15], *AtClAP* [16], *crypto-AC* [17], *NbAC* [18], *HpAC1* [19] and *MpCAPE* [20]. To investigate the sequence similarity of the eleven plant *ACs*, their sequences were aligned. The results showed that they have only 14.49% sequence similarity (Figure S1), indicating that *AC* genes in plants may be camouflaged under a wide range of large gene families and vary in their structure, expression, activity, and regulation.

Meanwhile, we used the ScanProsite tool (https://prosite.expasy.org/scanprosite (accessed on 9 May 2022))to reanalyze the catalytic center motifs of these plant ACs by the conserved consensus stringent motif [RKS]X[DE]X(9,11)[KR]X(1,3)[DE]. The results indicated that all the identified plant ACs contain the highly conserved consensus stringent motif, but harbor a different number of the catalytic center motifs (Table 1). Notably ZmPSiP, ZmRPP13-LK3, AtPPR-AC, AtLRRAC1, AtKUP5, MpCAPE, and AtKUP7, they possess six, four, three, three, three, three, and two distinct catalytic center motifs, respectively (Table 1). Specially, our group and one other have experimentally proved that each catalytic center motif of ZmPSiP, ZmRPP13-LK3 and AtLRRAC1belongs to functional AC [9,13].

According to above-mentioned search methods, previous AC searches and characterizations of catalytic center motifs in AC have excluded hits without [DE] [9,13,21]. Nevertheless, a recent work by Ruzvidzo et al. [22] showed that AC centers without the downstream [DE] residues could rescue the AC-deficient *E. coli* mutant strain SP850 with a cyaA mutation essential for lactose fermentation, but had significantly reduced activities compared to AC centers harboring the downstream [DE] residues in AtLRRAC1. Interestingly, we used the ScanProsite tool (https://prosite.expasy.org/ scanprosite (accessed on 9 May 2022)) to reanalyze the catalytic center motifs of these eleven known plant ACs by the conserved consensus stringent motif [RKS]X[DE]X(9,11)[KR]X(1,3). The results indicated that the number of the catalytic center motifs abundantly increase compared to that attained by catalytic center motif [RKS]X[DE]X(9,11)[KR]X(1,3)[DE] (Table 1) in ten ACs except HpAC1. For example, ZmPSiP increases an extra 9 motifs, and ZmRPP13-LK3 increases an extra 5 motifs. We hypothesize that multiple functional AC centers in respective protein adds to the complexity of regulating AC-cAMP signaling pathways in plant cells and may also result in a rapid initiation of cAMP signaling pathways by activating the catalytic center at the same time. This hypothesis will require further investigations in vitro and in vivo.

## 3. cAMP Mediates Heat Stress Response in Plants

Heat stress is an increasingly serious environmental stress for plants. Plants promote resilience by altering their cellular homeostasis and morphology under heat stress. Molecular processes underlying these responses have been intensively studied and found to encompass diverse mechanisms operating across a broad range of cellular components [7,9,23,24]. The accumulating experimental evidence suggests that cAMP regulates thermotolerance in plants, despite the fact that the identification of cAMP target proteins remains far behind that in animals [3,6].

### 3.1. Crosstalk between cAMP and Ca^2+^ Signal under Heat Stress Conditions

Ca^2+^ signaling is critical for regulating downstream responses in plants exposed to heat stress [10,25,26,27]. In *Arabidopsis* seedlings, it was demonstrated that a sudden transfer from 20 °C to 40 °C (7 min) could elevate the free Ca^2+^ concentration specifically in chloroplasts because a similar response was undetectable in the cytosol, indicating that chloroplasts use Ca^2+^ signals to call for help under heat stress [27,28]. On the contrary, in *Arabidopsis*, heat stress led to the increase of Ca^2+^ in the cytosol from 1 to 21 min when the temperature was increased from 22 to 37 °C [25].

When maize was exposed to heat stress, exogenous cAMP application obviously increased the expression of CSC1-like protein (Ca^2+^ transporter) and the uptake of Ca^2+^ in roots [24], as well as the expression of calmodulin protein 2 in leaves [9]; In tobacco BY-2 cells overexpressing the ‘cAMP-sponge’ as a genetic tool reducing intracellular cAMP levels (named as cAS cell), and cAMP deficiency significantly changed the expression of calcium-dependent lipid-binding (CaLB domain) family protein, annexin 2, calreticulin 3, calcineurin B-like 3, calcium-dependent phosphotriesterase superfamily protein, and calcium-binding EF-hand family protein under heat stress [7]. Interestingly, when *Arabidopsis* was subjected to heat stress, heat-increased AMP activated cyclic nucleotide-gated channel 6 (AtCNGC6) activity and thus resulted in an influx of Ca^2+^ into the cell via AtCNGC6, facilitating the expression of HSP genes and the acquisition of thermotolerance [25]; under the elevated cytosolic Ca^2+^ concentration, CaM2, CaM3, CaM5, and CaM7 negatively regulated Ca^2+^ conductivity of CNGC6 by binding its atypical isoleucine-glutamine motif, and thus led to a marked decrease in plasma membrane inward Ca^2+^ current, suggesting that the atypical isoleucine-glutamine motif plays an important role in CaM binding and the feedback regulation of the CNGC6 channel [29]. Taken together, these results indicate that cAMP-mediated Ca^2+^ signals could play a vital role in plant adaptation to heat stress, which linked plant heat perception to cytosolic cAMP elevation, a cAMP-activated Ca^2+^ channel and downstream heat stress response. Accordingly, considering the characteristics of multiple functional AC centers in respective AC protein, a hypothesis is proposed that an AC activity could act as membrane-associated temperature sensor rather than a Ca^2+^ channel [7,23,30].

In mammalian cells, ACs activity is sensitive to physiological relevant fluctuations in the CO_2_/HCO_3_^−^/pH, Ca^2+^ and ATP, especially in its substrate ATP. Thus, ACs functions as an environmental sensor and an integrator of intracellular signals (HCO_3_^−^, ATP or Ca^2+^) while tmACs respond to signals originating in other cells (i.e., hormones and neurotransmitters acting via GPCRs), and this is well summarized in reviews [5,31]. In maize, our group proved that two soluble ACs, ZmPSiP and ZmRPP13-LK3, are plasma membrane-attached protein and the mitochondria protein, respectively, and they were required for heat-induced cAMP synthesis and HSP expression [9]. However, it still isn’t clear whether the activities of ACs can be directly activated by the physiological fluctuations of heat-stimulated CO_2_/HCO_3_^−^/pH, Ca^2+^ and ATP in plant cells. To prove this it will be helpful to confirm whether the activities of ACs can act as membrane-associated temperature sensors.

### 3.2. cAMP Mediates H_2_O_2_ as Heat Stress Signals

Reactive oxygen species (ROS) as by-products of aerobic metabolism are key signaling molecules and play a significant role in the response of plants to a myriad of biotic and abiotic stresses [32]. The accumulation of ROS such as H_2_O_2_ is also a major cellular response to heat stress in plants. Heat-induced ROS originate mainly from the plasma membrane, chloroplasts, peroxisomes, and mitochondria [33]. When Arabidopsis protoplasts were subjected to a 45 s heat treatment at 42 °C, the accumulation of intracellular H_2_O_2_ was quickly stimulated, suggesting that H_2_O_2_ is involved in the early heat stress response and may function as the primary signaling molecules [34]. The respiratory burst oxidase homolog proteins (RBOHs) are plasma membrane-localized plant NADPH oxidases, which are important for generating ROS in the apoplastic space under abiotic or biotic stress [35]. It has been confirmed that the intracellular and extracellular content of ROS significantly increased in tobacco BY2 cells under a heat stress condition [7,36,37]. In maize leaves and roots, the application of cAMP analogous 8-Br-cAMP increased H_2_O_2_ content but had no influence on RBOHB expression under no heat stress, whereas prominently reduced H_2_O_2_ content but increased RBOHB expression under heat stress [9,24]. In tobacco cAS cells, cAMP deficiency resulted in a greater accumulation of RBOHC (A0A1S3YBQ2), which is consistent with the accumulation of intracellular ROS. In cases such as this, in mammalian cells, the reduction of cAMP content resulted in the higher expression of NADPH oxidase isoforms Nox1/Nox2/Nox4 and p47phox proteins and the enhancement of NADPH oxidase activity, which caused oxidative stress [38,39,40]. On the other hand, in human endothelial cells, the elevation of cellular cAMP increased Nox4 expression and H_2_O_2_ accumulation and prevented endothelial cell apoptosis [41]. Taken together, these results suggest that the complex relationships of cAMP with ROS accumulation depends on the species, conditions and RBOH isoforms.

In addition, recent data have revealed that Ca^2+^ could activate the RBOHB via CDPK7-mediated phosphorylation in maize exposed to heat stress [10]. However, the role of cAMP in this regulatory mechanism remains to be further determined.

ROS elimination is generally accompanied with increasing antioxidant enzymes, which is close to plant thermotolerance. In maize, the application of 8-Br-cAMP increased the activity of ascorbate peroxidase (APX) and superoxide dismutase (SOD), as well as the expression of glutathione S-transferase 4, GST6 protein, 14-3-3-like protein, 14-3-3-like protein GF14-12, and 14-3-3-like protein GF14-6 under heat stress [9,10]. In tobacco cAS cells under heat stress, the failure in cAMP elevation decreased activities of APX and catalase (CAT), as well as the accumulation of proteins involved in redox homeostasis [7]. Noticeably, the pathway by which cAMP stimulates the activity of enzymes to scavenge ROS as well as enhance plant thermotolerance has yet to be illuminated.

### 3.3. Involvement of cAMP in ABA Signaling Pathway under Heat Stress

The phytohormone abscisic acid (ABA) is a growth regulator that is involved in the adaptation to heat stress response by changing the adaptation process [9,24,42,43]. Emerging evidence has increasingly fit cAMP in the signaling pathways of ABA-dependent plant heat stress responses. Recent studies characterized that ZmRPP13-LK3 and ZmPSiP not only had AC activity but also involved ABA-mediated resistance to heat stress in maize [9,24]. Heat-enhanced cAMP content was decreased in maize ABA biosynthesis-deficient mutant viviparous-5 (*vp5*) comparable to its wild type Vp5, which is consistent with the change of heat-enhanced ZmRPP13-LK3 and ZmPSiP expression. Furthermore, 8-Br-cAMP pretreatment promoted the expression of WRKY106, RD29B, and ABA1 under heat stress, while there was no obvious effect on NCED3, SnRK2.2, AAO3, and P5CS1 [9]. Given that these marker genes are involved in the signal pathway and metabolism of ABA, it is proposed that cAMP is involved in the ABA-mediated-signaling pathway but not the ABA metabolism process. Therefore, these results implied the role of these two maize ACs in ABA-mediated thermotolerance [9,24].

Surprisingly, in *Arabidopsis*, the 9-cis-epoxycarotenoid dioxygenase (NCED3, At3g14440) as a key enzyme for the ABA synthesis, was identified as an AC and could be the first AC reported in the chloroplast of higher plants [17]. Thus, it is conceivable that during stress, the upregulation of NCED3 expression results in the content enhancement of ABA and cAMP, which could then mediate ABA signaling. ZmRPP13-LK3 and AtNCED3 are located in mitochondria and chloroplast, respectively. Therefore, characterizing AC-dependent downstream molecular processes and physiological responses may provide opportunities of organelle-level manipulations to generate crops with elevated tolerance to abiotic stresses [17].

In addition to regulating physiological functions in plants, ABA is also produced and released by several mammalian cell types, including human granulocytes, where it stimulates innate immune functions via an increase of the intracellular cAMP concentration [44,45]. The lanthionine synthetase C-like protein LANCL2, an ABA receptor in mammalian cells, is a peripheral membrane protein localizing at the intracellular side of the plasma membrane. It has been reported that ABA could stimulate ATP release through the LANCL2-mediated activation of AC [45,46]. These findings are potentially linking cAMP to ABA signal pathways in the resistance against stress.

### 3.4. cAMP and HSP under Heat Stress

In plant response to heat stress, the expression of heat shock proteins (HSPs) will rapidly increase via a heat shock factor (HSF)-dependent mechanism. Heat stress results in the misfolding and denaturation of proteins. Misfolded proteins are highly toxic because they can form aggregates and hamper the normal activities of cells. HSPs participating in protein folding and assembly play an indispensable role in plant tolerance to heat stress [47].

Emerging evidence indicates that the heat-mediated increase of cAMP level is positively correlated to the expression of HSFs and HSP in plants [7,9,24,25]. In maize leaves or roots, heat stress significantly upregulated the expression of *HSPs* which belong to *HSP90*, *HSP70* and *HSP20* families. Moreover, cAMP pretreatment further promoted heat-upregulated expression of HSPs; RNAi against two maize *AC* genes-*ZmRPP13-LK3* and *ZmPSiP* significantly reduced the content of cAMP and the expression of several HSPs [9,10]. In Arabidopsis response to heat stress, heat-increased cAMP mediated Ca^2+^ influx by activating the activity of CNGC6; the concentration of Ca^2+^, the expression of *HSP* genes, and the acquisition of thermotolerance were abundantly decreased and increased in *cngc6* knockdown lines and CNGC6 over-expression lines, respectively; the treatment with exogenous cAMP analogue obviously enhanced the expression of *HSP* genes under heat stress conditions [25]. These data indicate that cAMP upregulates the expression of *HSP* genes via Ca^2+^-activated CNGC6 activity under heat stress conditions.

Nevertheless, the above-mentioned data are apparently in conflict with the work of Paradiso et al. [7], who reported that heat stress enhanced the expression of *HSPs* and the content of cAMP in tobacco BY-2 cells, but the extent of heat-increased *HSP18*, *HSP26* and *HSP101* expression were similar in cAMP-deficient cAS and WT cells of tobacco BY-2 cells, suggesting that heat stress-dependent cAMP elevation is not needed for heat stress-increased HSPs expression in tobacco BY-2 cells [7]. The possible reasons for the divergence are: (1) different experimental material: Paradiso et al. [7] used tobacco BY-2 cells exposed to heat stress, whereas other groups used the intact maize or Arabidopsis plants exposed to heat stress [9,10,25]; (2) the ability of cAMP sponge to sequester cAMP was up to saturation state after five days of heat stress treatment, and for the time being the content of cAMP in cAS cells was similar to that in WT cells of tobacco BY-2 cells [7]; (3) heat stress increased the expression of *HSPs* possibly by a cAMP-dependent and independent way.

The heat-induced increase in the expression of *HSPs* was significantly greater in cAS cells than that in WT cells of tobacco BY-2 cells under heat stress. It might be that the cAMP-deficient cAS cells were more sensitive than WT under heat stress and hence needed higher *HSP* expression to maintain protein function [7]. A similar phenomenon is found in the maize ABA-deficient mutant *vp5* and its wild-type Vp5 [9]. These results indicated that cAMP is integrated into the complex heat stress network in plants, and heat stress induced the expression of *HSPs,* possibly in a cAMP-dependent and -independent way.

### 3.5. cAMP Mediates the Trafficking of Vesicle and Biomacromolecules under Heat Stress

Vesicle trafficking is a fundamental cellular process in all eukaryotic cells and is tightly linked to stress-related signaling pathways to meet the demands of rapid changes in cellular processes as well as to ensure the correct delivery of stress-related cargo molecules [48]. For example, transport protein particle (TRAPP) complexes play a key role in the selective delivery of membrane vesicles to various subcellular compartments [49]. When tobacco BY-2 cells are exposed to heat stress, the expression of a TRAPP component was significantly lower in cAMP-deficient cAS cells than that in WT [7], indicating cAMP might accelerate the selective delivery of membrane vesicles to various subcellular compartments. In maize, 8h heat stress did not significantly affect the expression of eight vesicle transport-related proteins, including vacuolar protein sorting-associated protein 54, vacuolar protein sorting-associated protein 32 homolog 1, vacuolar protein sorting-associated protein 2 homolog 1, SNAP25 homologous protein SNAP33, charged multivesicular body protein 5, syntaxin-41, and SynN domain-containing protein, but heat stress following cAMP application obviously increased their expression compared to without pretreatment. These results indicate that cAMP might speed up the vesicle trafficking for turnover of cargo molecules [9,24].

By the way, nuclear transport factor 2 (NTF2) is one of essential components in nuclear trafficking [50]. When tobacco BY-2 cells are exposed to heat stress, the expression of two NTF2s were significantly lower in cAMP-deficient cAS cells than that in WT [7], indicating that cAMP might accelerate the exchange of nucleocytoplasmic biomacromolecules.

### 3.6. cAMP Participating in Ubiquitin-Proteasome System (UPS) under Heat Stress

The UPS provides a rapid and efficient strategy to control many different cellular processes by selectively removing the regulatory proteins, and thus plays a critical role in regulating a wide range of cytological and physiological processes in plant and mammalian cells. Besides the lysosome (lytic vacuole of plant cells) and the proteases of cytosol and mitochondrion, the UPS is the major protein degradation pathway responsible for the degradation of 80–90% proteins. Increasing evidence indicates that the UPS is also an integral part of plant adaptation to environmental stimuli, such as nutrient deprivation, drought, cold, salinity, heat stress and pathogens (well reviewed in: Xu et al. [51] and Vierstra [52]).

Due to protein aggregation under heat stress, plant survival to proteotoxic stress requires the coordination of disaggregation and refolding of proteins and proteolysis [53]. In *Arabidopsis*, bioinformation analysis indicated that more than 1600 genes encode UPS-related factors, including over 1400 E3s [52]. The particular emphasis on this proteolytic system in plants may be due to the long-life spans and sessile habit that require effective metabolism regulation to better survive environmental stress.

In tobacco BY2 cells, the protease activity under control conditions had no significant differences between WT and cAMP-deficient cAS cells, whereas proteasome activity in cAS cells was obviously lower than that in WT cells, indicating the proteasome has already been impaired under control conditions. Moreover, the heat stress-caused increase of protease and proteasome activity was only in WT cells. Consistently, cAMP-deficient cAS cells under heat stress failed to accumulate fifteen UPS-related proteins, including 20S core particle and non-ATPase regulatory particles as well as proteins of ubiquitin family [7]. Similarity, a recent study in maize revealed that heat stress did not affect the expression of many UPS-related proteins, whereas cAMP pretreatment significantly promoted the expression of these proteins, including ubiquitin-like-specific protease ESD4, subtilisin-like protease SBT2.6, ATP-dependent Clp protease ATP-binding subunit CLPT1 chloroplastic, 26S proteasome non-ATPase regulatory subunit 12 homolog B, ubiquitin-activating enzyme E1, 26S proteasome non-ATPase regulatory subunit 4-like protein, ubiquitinyl hydrolase 1, and RING-type E3 ubiquitin transferase [9,24].

Taken together, these results suggest that the avoidance of proteotoxic stress could be a key step of heat stress response. The cAMP-mediated selected-protein degradation may be an important plant strategy to deal with environmental stress. Nevertheless, the precise functions of cAMP in UPS pathway under heat stress still needs further investigation, which will provide informative hints on the regulation of UPS components and proteasome activity via the cAMP signaling pathway under environmental stimuli.

## 4. Future Perspectives

Heat stress disturbs cellular homeostasis and impedes the growth and development of plants, which brings about extensive agricultural losses and threatens the sustainability of agricultural production. Plants have evolved a variety of thermotolerant mechanisms to minimize damage and protect themselves from further heat stress. Sensing and response to heat stress are crucial to prevent heat-caused damage and preserve metabolic and cellular functions. In higher plants, the identification of functional diversified ACs and the validation of a cAMP-dependent signaling system have spurred great scientific interest on the polyhedral role of cAMP regulating physiological processes and the stress response of plants [6,7,9,17,24].

cAMP is increasingly recognized as an important signaling molecule in plant response to heat stress and is involved in multi-level regulatory networks to avoid cell damage and maintain cellular homeostasis (Supplementary Table S1) [7,9,17,24]. Heat stress prominently induced the expression of two maize ACs-ZmPSiP and ZmRPP13-LK3 as well as the accumulation of cAMP [7,9,24,25]. Heat-increased cAMP regulated Ca^2+^ influx via the CNGC6 channel, which further promoted the expression of HSPs [25]. In addition, cAMP upregulated the expression of UPS components and the activity of 26s proteasome under heat stress [7,9,10]. These results indicate that HSPs and UPS function in the downstream of the heat-caused cAMP signaling pathway. In animal cells, the study demonstrated that inhibition of the ubiquitin proteasome induced the expression of cytosolic HSPs under heat stress. Both proteasomes and chaperones jointly act on the removal of aberrant proteins to maintain protein homeostasis [54,55]. Nevertheless, the mechanism of cAMP regulating the cooperative linkage between chaperone and UPS pathways remains largely elusive so far in both animal and plant cells.

Moreover, the clear identification and functional characterization of cAMP-binding proteins (CNBPs) and direct regulators of ACs participating in the ACs-cAMP signaling cascade are required. In *Arabidopsis*, 15 candidate CNBPs have been identified by an affinity purification technique. Among them, seven function in the photosynthesis or photorespiration pathway, one participates in chlorophyll synthesis, one is involved in vesicle transport from the endoplasmic reticulum to the Golgi apparatus, one is associated with the chloroplast protein import apparatus as a molecular chaperone, two function during protein biosynthesis, and one participates in the chloroplast ribosomal RNA metabolism [56]. Nevertheless, the complex architecture of cAMP-dependent pathways is far from being fully understood because upstream actors of AC and downstream binding proteins of cAMP remain largely unidentified, especially under heat stress. New bioinformatics and molecular tools will provide opportunities to extend our presently scarce knowledge.

The involvement of cAMP in plant thermotolerance was supported by the identification of maize ACs involved in plant response to heat stress [9,24]. K^+^-uptake permease AtKUP7 and AtKUP5 in *Arabidopsis* have been proved to be ACs. K^+^ uptake via AtKUP5 can stimulate self AC activity to generate more cAMP [14,15]. K^+^ as an important osmolyte is involved in turgor-dependent volume regulation, such as the stomatal movement, which is a vital biological process in higher plant response to stress. Therefore, the function of both AtKUP7 and AtKUP5 in plant response to heat stress is worth clarifying. In addition, the water retention ability (relative water content) was observed to be negatively correlated with exposure to heat stress [57]; the addition of 8-Br-cAMP completely reversed exogenous ABA- and Ca^2+^-induced inhibition of whole-cell inward K^+^ currents and the stomatal opening in *Vicia faba* [58]. The effect of cAMP application on the stomatal opening, transpiration/evaporation loss and plant productivity in plants exposed to heat stress needs to be investigated further.

**Figure 1:**
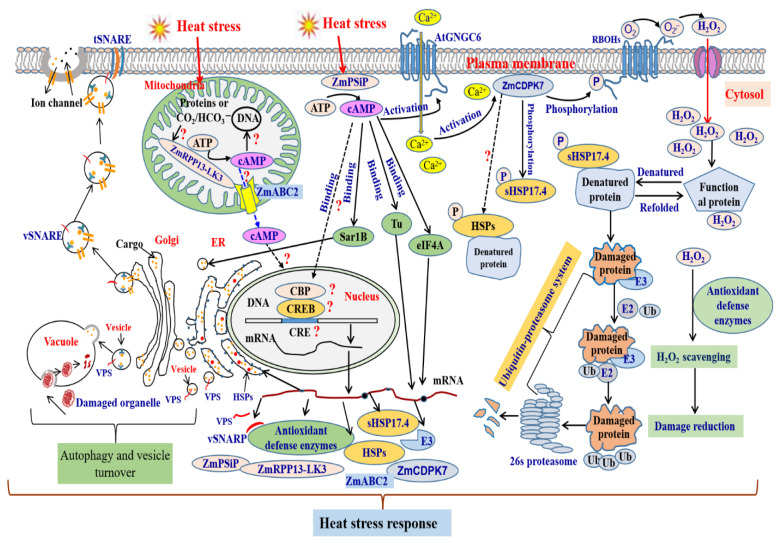
A proposed model of cAMP signaling pathways in plant cell response to heat stress. The figure draws on actual knowledge about the AC-cAMP signaling cascade in plants under heat stress. ATP, adenosine triphosphate; cAMP, cyclic adenosine monophosphate; CBP, cAMP binding protein; CRE, cAMP response element; CREB, CRE binding protein. CNGC, cyclic nucleotide gated channel; SNARE: including SNAP25 homologous protein SNAP33, Syntaxin-41, and SynN domain-containing protein; VPS: including vacuolar protein sorting-associated protein 32 homolog 1, vacuolar protein sorting-associated protein 32 homolog 1; ubiquitin-proteasome system: including ubiquitinyl hydrolase 1, protease, 26S proteasome non-ATPase regulatory subunit and so on. The heat-upregulated candidates are depicted in blue font. This figure draws on recent studies of tobacco BY-2 cell, Arabidopsis thaliana, and maize cAMP roles under heat stress [7,9,10,24,25].

Although upstream and downstream actors of the AC-cAMP signaling cascade still await detailed characterization, we propose a model on possible regulatory mechanisms in plant cell response to heat stress (Figure 1). In this model, cAMP is generated by ZmPSiP and ZmRPP13-LK3 under heat stress [9,24]. AtCNGC6 is the identified target of cAMP modulating the opening/closing of CNGC6 pores by direct binding to the cyclic nucleotide binding domain, which caused Ca^2+^ flux in plant cell response to heat stress [25]. The heat-induced Ca^2+^ increase stimulated the kinase activity of ZmCDPK7. Thereafter, ZmCDPK7 phosphorated sHSP17.4 and RBOH, which possibly activated sHSP17.4’s chaperone role for restoring the function of denatured proteins and activated the RBOH enzyme activity to generate ROS. ROS accumulation further caused the enhancement of antioxidant enzyme activity to reduce ROS accumulation [9,10,24]. Meanwhile, cAMP also regulated the expression of various genes related to vesicle formation and transport, UPS pathway, and mRNA translation initiation. Additionally, cAMP possibly regulated the process of mRNA translation initiation, vesicle formation and transport by directly binding target proteins such as eukaryotic translation initiation factor 4A1, elongation factor Tu, and GTP-binding protein SAR1B [7,9,10,55]. The comprehensive results indicated that cAMP was involved in cell homeostasis by regulating heat response gene expression, various ion transporters, protein degradation via the ubiquitin-proteasome system or autophagy, a rapid delivery of stress-related cargo molecules by regulating vesicle trafficking, and HSPs-assisted protein processing in ER and cytosol [7,9,10,24]. Nevertheless, it still requires the investigation of whether the heat-increased cAMP can initiate downstream signal transduction cascades acting on protein kinases that in turn phosphorylate transcriptional factors to fine tune cell homeostasis in plants (Figure 1).

## Figures and Tables

**Table 1:** The analysis of catalytic center motifs of eleven known plant ACs.

Organism	Protein Name	Accession Numbers	Catalytic Center Motif by [RKS]X[DE]X(9,11)[KR]X(1,3)[DE]	Extra Catalytic Center Motif by [RKS]X[DE]X(9,11)[KR]X(1,3)
*Zea mays*	Putative disease resistance RPP13-like protein 3 (ZmRPP13-LK3)	A0A1D6NWF3	K_174_VDNARKMMTEKEEKKIWE_192_	S_23_TEERMKKLFGDFEK_37_
			K_185_EEKKIWEDQKAKELEE_201_	S_539_GEVSLPKDIHQMR_552_
			S_498_LERGVGTTTRKLRTLLD_515_	S_585_IDHIPASLWRNR_597_
			S_69_GDQVGVIGFDEQIKQIE_86_	S_707_NDHKILELGRIK_719_
				S_820_DDEKVFQHLPIWR_833_
*Zea mays*	Pollen-Signaling Protein (ZmPSiP)	AJ307886	S_146_DDAKRDWFSRDVCKNCSD_164_	R_43_IDAIISHEERRR_55_
			S_346_EELATLKDVGIKIAE_361_	S_155_RDVCKNCSDAMK_167_
			S_698_LDRATSGASALANKPFLE_716_	K_193_VDVFAIVGAVGIGK_207_
			R_115_HEIGFTIRDIDLRLRE_131_	K_259_EELLILLASALSKR_273_
			K_741_EEKEGQERSNGQCRGDE_758_	S_482_VERCWITHHLLR_494_
			S_1049_CDGKRYFRYNKSRRIYE_1066_	S_528_MENSLDGPISLK_540_
				S_532_LDGPISLKQQMGLR_546_
				K_736_DETEKEEKEGQER_749_
				S_933_DELHLKDNKVLQR_946_
*Arabidopsis thaliana*	LRR and NB-ARC domains-containing disease resistance protein (AtLRRAC1)	At3g14460	K_121_MEKVVRLLEHHVKHIE_137_	R_310_SEIVSTVAKAEK_322_
			R_1302_I EWGLRDLENLRNLE_1317_	R_505_LEDDNIPEIPSTTR_519_
			R_159_PDDLPQGRLVGRVED_174_	S_1128_LESFPGSHPPTTLK_1142_
				S_1377_IDEDLPPLSCLR_1389_
*Arabidopsis thaliana*	Pentatricopeptide (PPR) repeat-containing protein (AtPPR-AC1)	AT1G62590-	K_100_FDVVISLGEKMQRLE_115_	K_65_LDDAIGLFGGMVK_78_
			R_238_GDTDLALNLLNKME_252_	R_380_LDKAKQMFEFMVSK_394_
			K_485_LEKALEVFDYMQKSE_500_	R_415_VEDGTELFREMSHR_429_
				K_520_VDDGWDLFCSLSLK_534_
*Arabidopsis thaliana*	K^+^ uptake permease 7 (AtKUP7)	AT5G09400	S_80_FDVEALEVPGAPRNDYE_97_	S_61_DEDEIPEHRLIR_73_
			S_697_LEKFIRREAQERSLE_712_	S_774_SDSSVSEAEQSLER_788_
				S_785_LERELSFIHKAK_797_
*Arabidopsis thaliana*	K^+^ uptake permease 5 (AtKUP5)	AT4G33530	S_81_FDVDALEIPGTQKNEIE_98_	S_61_DEEDDNVEQRLIR_74_
			S_212_PELERSLIIKERLE_226_	S_782_LEKELSFIHKAK_794_
			S_698_LEKFIRKEAQERALE_713_	
*Arabidopsis thaliana*	Clathrin assembly protein (AtClAP)	AT1G68110	K_329_WEIFEDDYRCFDRKDKWE_347_	S_43_HDDSSVDYSNAHR_56_
				S_48_VDYSNAHRVYKWIR_62_
				S_165_LEKTSDSVIQELER_179_
				K_236_SEAATVLKIVNK_248_
				K_307_EDEKAMVVLEQPKK_321_
*Hippeastrum hybrid cultivar*	Adenylate cyclase (HpAC1)	ADM83595	S_160_YEIECETTEPERVKGLLE_178_	
*Marchantia polymorpha*	Hypothetical protein MARPO_0068s0004 (MpCAPE)	PTQ35772	K_486_GEAVQEGYQQDHSKLE_502_	K_7_DDKGKDQEENDEAK_21_
			K_788_AEPETEDEYNQRYE_802_	S_326_SDAPPSPKRLPDK_339_
			R_800_YEAPLSLLFGAPTREE_816_	S_597_QEQRGSPSPGVQYR_611_
				R_814_EELMLPAKIGKDK_827_
				S_1053_PDYEEEEKFLSK_1065_
				S_1242_RDSNRLLIQPIER_1255_
				S_1419_IETSQSLAEFAK_1431_
*Nicotiana benthamiana*	Adenylyl cyclase (NbAC)	ACR77530	R_354_LEVIKRQKDEKRKE_368_	R_135_EDVNFLHDILQRLR_149_
*Arabidopsis thaliana*	Nine-cis-epoxycaroteoid dioygenase 3 (crypto-AC)		S_311_YDVVSKPYLKYFRFSPD_328_	S_29_SDLSYCSSLPMASR_43_
				S_304_GELFALSYDVVSK_317_
				S_441_DENLKSVLSEIR_453_
				S_567_LEVEATVKLPSR_579_

Notes: The catalytic center motif covered by a gray shadow has been reported on in a published paper. ScanProsite tool: https://prosite.expasy.org/scanprosite (accessed on 8 June 2022).

## Data Availability

Not applicable.

## Supplementary Materials

The following supporting information can be downloaded at: https://www.mdpi.com/article/10.3390/life12060885/s1, Figure S1: Sequence alignment of the adenylyl cyclases (ACs) identified in plants; Table S1: cAMP-mediated biological processes under heat stress. References [7,9,10,59] are cited in the supplementary materials.
