# Supplementary material for: cAMP Is a Promising Regulatory Molecule for Plant Adaptation to Heat Stress
Source: Life (Basel). 2022 Jun 14;12(6):885. doi: 10.3390/life12060885 (PMC9225146; doi:10.3390/life12060885)
Supplement: Supplementary file 1 [file life-12-00885-s001.zip › Figure S1.pdf]

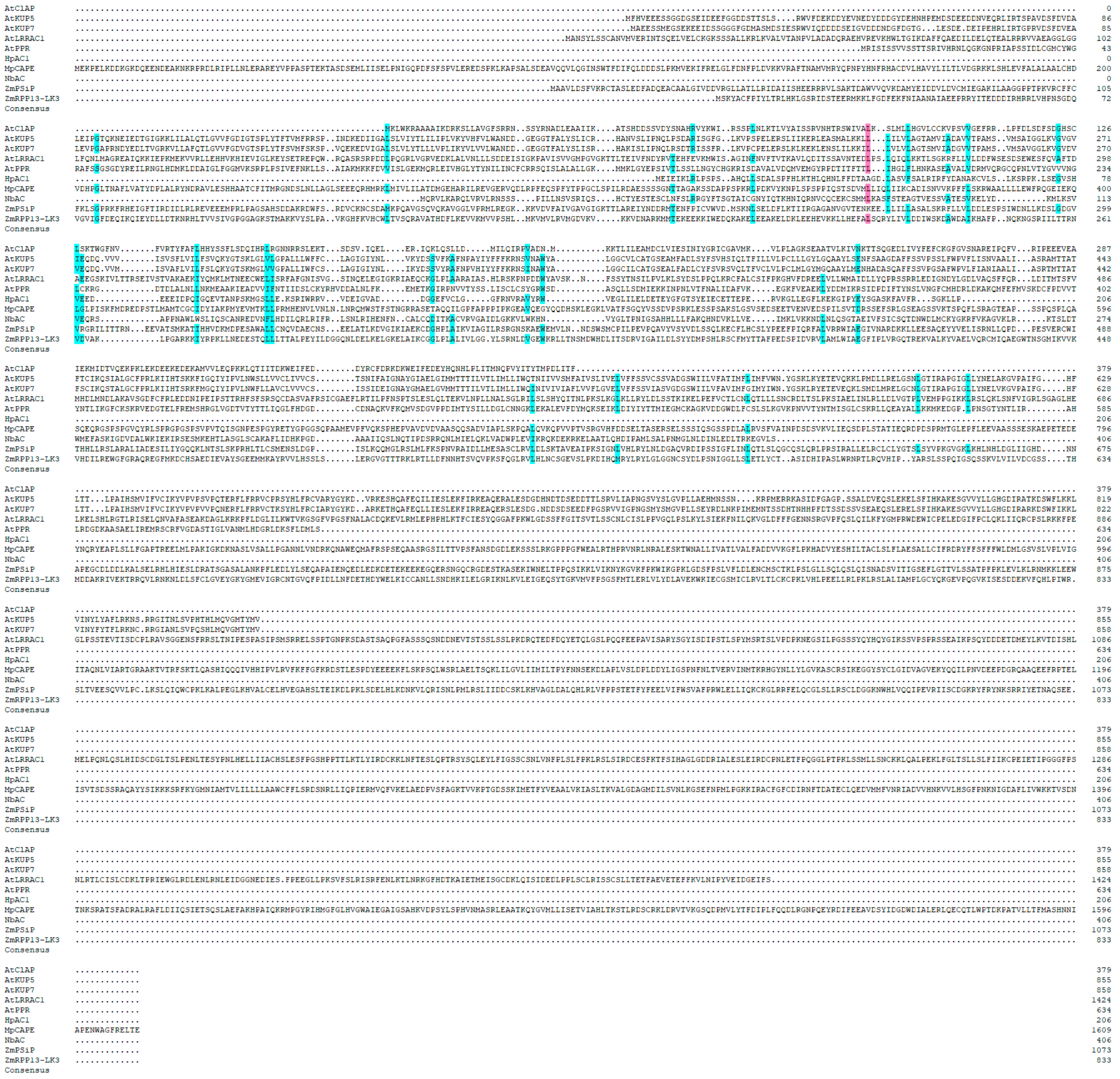

Figure.S1 Sequence alignment of the adenylyl cyclases (ACs) identified in plants. Blue and red indicates 70-90% and 50-70% sequence similarity among the elven identified ACs in plants, respectively.
