# Supplementary material for: cAMP Is a Promising Regulatory Molecule for Plant Adaptation to Heat Stress
Source: Life (Basel). 2022 Jun 14;12(6):885. doi: 10.3390/life12060885 (PMC9225146; doi:10.3390/life12060885)
Supplement: Supplementary file 1 [file life-12-00885-s001.zip › Table S1.pdf]

Table S1. cAMP-mediated biological processes under heat stress

Biological Processes	References
Cellular calcium ion homeostasis	[1–4]
Chaperone-mediated protein folding	[1–4]
Chloroplasts-associated	[2–4]
Cytoskeleton organization	[1–4]
Defense response, incompatible interaction	[2–4]
Detection of biotic stimulus	[1–4]
Hydrogen peroxide metabolic process	[1–4]
Ion transport and cell signaling	[1–4]
Lipid oxidation	[2–4]
Macromolecular complex assembly	[2–4]
Macromolecule metabolic process	[2–4]
Malate metabolic process	[2–4]
Membrane fusion	[2–4]
Mitochondria-associated	[2–4]
mRNA metabolic process	[2–4]
Phytohormone biosynthesis and homeostasis	[2–4]
Proteasomal degradation	[2–4]
Protein metabolic process	[2–4]
Protein phosphorylation	[2–4]
Regulation of cell death	[2–4]
Response to hormone (abscisic acid, auxin, salicylic acid)	[2–4]
Small GTPase mediated signal transduction	[2–4]
Transcription and translation	[2–4]
Vacuolar transport	[2–4]

References

1. Gao, F.; Han, X.; Wu, J.; Zheng, S.; Shang, Z.; Sun, D.; Zhou, R.; Li, B. A heat-activated calcium-permeable channel—Arabidopsis cyclic nucleotide-gated ion channel 6—is involved in heat shock responses. *Plant J.* **2012**, *70*, 1056–1069.
2. Paradiso, A.; Domingo, G.; Blanco, E.; Buscaglia, A.; Fortunato, S.; Marsoni, M.; Scarcia, P.; Caretto, S.; Vannini, C.; de Pinto, M.C. Cyclic AMP mediates heat stress response by the control of redox homeostasis and ubiquitin-proteasome system. *Plant Cell Environ*. **2020**, *43*, 2727–2742.
3. Yang, H.; Zhao, Y.; Chen, N.; Liu, Y.; Yang, S.; Du, H.; Wang, W.; Wu, J.; Tai, F.; Chen, F.; et al. A new adenylyl cyclase, putative disease resistance RPP13-like protein 3, participates in abscisic acid-mediated heat stress resistance in maize. *J. Exp. Bot.* **2021**, *72*, 283–301.
4. Zhao, Y.L.; Du, H.W.; Wang, Y.K.; Wang, H.L.; Yang, S.Y.; Li, C.H.; Chen, N.; Yang, H.; Zhang, Y.H.; Zhu, Y.L.; et al. The calcium-dependent protein kinase ZmCDPK7 functions in heat-stress tolerance in maize. *J. Integr. Plant Biol.* **2021**, *63*, 510–527.
